# Postoperative water and electrolyte disturbances after extended endoscopic endonasal transsphenoidal surgery

**DOI:** 10.3389/fendo.2022.963707

**Published:** 2022-08-23

**Authors:** Juan Manuel Canelo Moreno, Elena Dios Fuentes, Eva Venegas Moreno, Pablo Jesús Remón Ruíz, Cristina Muñoz Gómez, Ana Piñar Gutiérrez, Eugenio Cárdenas Valdepeñas, Ariel Kaen, Alfonso Soto Moreno

**Affiliations:** ^1^ Unidad de Gestión Clínica de Endocrinología y Nutrición, Instituto de Biomedicina de Sevilla (IBIS), Virgen del Rocio University Hospital, Centro Superior de Investigaciones Científicas (CSIC), University of Seville, Seville, Spain; ^2^ Unidad de Gestión Clínica de Neurocirugía, Virgen del Rocío University Hospital, Seville, Spain

**Keywords:** pituitary adenoma, transsphenoidal surgery, extended endoscopic endonasal approach, polyuria, diabetes insipidus

## Abstract

**Introduction:**

Water and electrolyte disturbances are common after pituitary surgery and can generally be classified into transient hypotonic polyuria and transient or permanent diabetes insipidus (DI). The prevalence varies in the literature between 31-51% for transient hypotonic polyuria, 5.1-25.2% for transient DI, and 1-8.8% for permanent DI.

**Objective:**

The aim of this study was to identify the prevalence of water and electrolyte disturbances with polyuria and the preoperative and postoperative predictive factors in patients undergoing surgery with an extended endoscopic endonasal approach.

**Material and methods:**

This retrospective observational descriptive study included 203 patients with a diagnosis of pituitary adenoma who underwent their first transsphenoidal surgery *via* the extended endoscopic endonasal approach between April 2013 and February 2020. The diagnosis of water and electrolyte disturbances was based on the criterion for polyuria (>4 ml/kg/h). Postoperative polyuria was defined as those cases diagnosed during the immediate postsurgical period that resolved prior to discharge. Transient DI included all cases with a duration of less than 6 months but still present at hospital discharge, and permanent DI included cases lasting more than 6 months.

**Results:**

The overall prevalence of water and electrolyte disorders was 30.5% (62), and the prevalence of postoperative polyuria was 23.6% (48). The median number of desmopressin doses administered to patients with postoperative polyuria was one dose (interquartile range [IQR] 1-2), and thus the median duration of treatment was 0 days. The median initiation of desmopressin was the second day after surgery (IQR 1-2). The overall prevalence of DI was 6.89%. Among the patients with transient DI, the duration was less than 3 months in three patients (1.47%), and between 3 and 6 months in two (0.98%). Nine patients had permanent DI (4.43%). (4.43%).

**Conclusions:**

The prevalence of electrolyte disturbances in our study was high, although similar to that found in the literature. Most of the cases were transient hypotonic polyuria that resolved within one day. The prevalence of transient DI in our cohort was lower than that described in the literature, while permanent DI was similar.

## Introduction

Transsphenoidal surgery is a procedure to access pituitary tumors or sellar masses. One of the most common side effects after surgery is altered water and electrolyte homeostasis due to manipulation of the posterior pituitary and infundibulum (1). Polyuric water and electrolyte disturbances can generally be categorized as transient hypotonic polyuria and transient or permanent diabetes insipidus (DI). The prevalence varies in the literature between 31-51% for transient hypotonic polyuria, 5.1-25.2% for transient DI, and 1-8.8% for permanent DI (2).

Polyuria following pituitary surgery can be caused by a physiological response to excess fluid, but may also be a manifestation of osmotic diuresis or DI (1). DI is part of the syndrome termed polyuria-polydipsia and is characterized by hypotonic polyuria (excessive urine output > 50 ml/kg body weight in 24h), polydipsia (excessive fluid intake > 3L/day), and the exclusion of other osmotic diuresis disorders. The differential diagnosis includes both primary forms (central or renal origin) and secondary forms (primary polydipsia). While in nephrogenic DI the genetic causes are the most frequent, in central DI the causes are acquired: due to alterations produced in the neurohypophysis by damage to the magnocellular nuclei that produce arginine vasopressin (AVP) (3, 4).

Although water and electrolyte disturbances occur acutely, no reliable risk factors or markers have been established to predict the onset of these disorders (1, 5). Only a limited number of studies have analyzed the clinical factors for postoperative DI, with inconsistent results (6). While in some studies a higher risk is associated with Rathke’s cysts or larger adenomas (7, 8), others do not confirm these findings (9). On the other hand, DI has been associated with higher rates of postoperative morbidity and hospital stay (6).

Given the frequency of water and electrolyte disturbances following pituitary surgery, and the lack of knowledge concerning the risk factors, the aim of this study was to identify the prevalence of water and electrolyte disturbances with polyuria and the predictive preoperative and postoperative factors in patients undergoing surgery with an extended endoscopic endonasal approach.

## Material and methods

This single-center retrospective descriptive observational study included 203 patients with a diagnosis of pituitary adenoma in whom transsphenoidal surgery was performed using the extended endoscopic endonasal approach (EEEA) between April 2013 and February 2020 at the Virgen del Rocio University Hospital in Seville, Spain.

Inclusion criteria comprised patients with pituitary adenoma who underwent surgery for the first time by EEEA, for whom clinical data were available from their digital health record. Patients who had already undergone previous EEEA surgery were excluded. Similarly, patients with pituitary tumors that did not correspond to adenomas, such as Rathke’s cleft cysts or craniopharyngiomas, were excluded. All patients with previous DI were excluded.

The diagnosis of water and electrolyte disturbance was made based on the criterion for polyuria (>4 ml/kg/h) for two consecutive hours. Patients with postoperative polyuria were those diagnosed in the immediate postoperative period with resolution prior to hospital discharge. Patients with transient DI included those with DI duration less than 6 months, which was still present at hospital discharge. Patients with permanent DI were those with DI lasting more than 6 months. Resolution of the condition was determined by withdrawal of desmopressin and evaluation of water metabolism. The criterion for administration of subcutaneous desmopressin was the same as at diagnosis, polyuria (>4 ml/kg/h) for two consecutive hours, which could be repeated after 8 hours if necessary.

The study variables were the following: age at baseline and age at diagnosis, duration of hospital stay, maximum size of the adenoma determined by magnetic resonance imaging of the sella turcica, sex, functional type determined by hormone production, and the presence of any previous visual deficit, determined by campimetry, attributable to the pituitary lesion. Surgical complications included sepsis, post-surgical meningitis, cerebrospinal fluid fistulas, tumor bleeding, or hydrocephalus. Cavernous sinus invasion was evaluated using the KNOSP classification, determining as invasive those with a KNOSP classification ≥ 3. The qualitative assessment of size distinguished between microadenoma smaller than 10 mm and macroadenoma larger than 10 mm, as well as between tumors larger and smaller than 30 mm. The following were studied in postoperative polyuria: number of doses of desmopressin and duration of treatment. Sodium levels before and after the first dose of desmopressin in mEq/L were assessed for all patients with water and electrolyte disorders.

Statistical analyses were performed with SPSS version 25. In the descriptive analysis, categorical variables are expressed as percentages with absolute values. Quantitative variables are expressed as mean ± standard deviation for those with a normal distribution and as median and interquartile range for those with a non-normal distribution. The assumption of normality was carried out using the Kolmogorov-Smirnoff test. For quantitative variables with a normal distribution, Student’s *t*-test was performed for independent samples, while for those with non-normal distribution, the Mann-Whitney U test was used. For categorical variables, the Chi-square test was used, except for those with a value ≤ 5 in any cell of the contingency table, in which Fisher’s test was used. In all cases, a value of p < 0.05 was considered statistically significant.

## Results

### Baseline characteristics

The overall prevalence of water and electrolyte disorders was 30.5% (62 patients). Of the 203 patients included in the study, 111 (54.7%) were women. The median age at diagnosis was 53 years (IQR 40-62). The median length of stay was 6 days (IQR 4-7).

Of the total cohort, 115 (56.7%) had non-functioning adenomas, 44 (21.7%) had ACTH- secreting tumors, and 44 (21.7%) had GH-secreting tumors. Previous visual deficit was present in 84 (42.6%) patients. Ninety-one patients had a previous hormonal deficit. Concerning invasiveness as measured by the Knosp classification, 78 (38.4%) patients had an invasive Knosp grade. The mean size of the adenomas was 22.05 ± 12.25 cm., and 79.9% of the adenomas were macroadenomas **Table 1**.

**Table 1:** Baseline characteristics.

	Total (N-203), n (%)
Age at baseline (years) median (IQR)	57 (45-67)
Age at diagnosis (years) median (IQR)	53 (40-62)
Hospital stay for surgery (days) median (IQR)	6 (4-7)
Maximum size (mm) median (SD)	22,05 ± 12,25
Sex	
Male	92 (45,3)
Female	111 (54,7)
Functional type	
Non-functioning	115 (56,7)
ACTH	44 (21,7)
GH	44 (21,7)
Previous visual deficit	
Yes	84 (42,6)
No	113 (57,4)
NA	6
Invasive^1^	
Yes	78 (38,4)
No	125 (61,6)
Size^2^	
Microadenoma	40 (20,1)
Macroadenoma	159 (79,9)
NA	4

IQR, Interquartile range.

SD, Standard deviation.

NA, Data not available.

1 Determined as invasive if it has a KNOSP rating of 3 or higher.

2. Macroadenoma size ≥ 10 mm.

### Levels of natremia

The mean sodium level prior to the first desmopressin administration was 140.41 mEq/L +/- 4.3. Of the 62 patients in whom desmopressin treatment was initiated, 8.1% presented a level at the lower limit of normality (ranging 133-134 mEq/L), 82.1% had eunatremia and six (9.7%) patients had hypernatremia, three of whom subsequently had permanent DI. The mean sodium level after the first dose of desmopressin was 140.61 mEq/L +/- 6.13. After desmopressin administration, 41 patients (67.2%) were eunatremic, and nine (14.8%) developed hypernatremia. Eleven patients (18%) had hyponatremia, although only four patients had a natremia level < 130 mEq/L. None of the patients with a subsequent diagnosis of DI had hyponatremia after the first dose of desmopressin.

### Postoperative polyuria

The prevalence of postoperative polyuria was 23.6% (48 patients). The median number of doses of desmopressin administered in patients with postoperative polyuria was one dose (IQR 1-2). In 30 patients, only one dose was administered, two doses in 14 patients, three doses in three patients, and four doses in one patient. The median duration of treatment was 0 days. The median initiation of desmopressin was the second day after surgery (IQR 1-2). Similarly, the median of the last dose administered was the second day after surgery (IQR 2-2.75).

In the comparative analysis of patients with postoperative polyuria versus those without water and electrolyte disturbances, Differences were found in age at baseline or age at diagnosis, median age 53 vs 58 years and 49 vs 54 years, respectively. There were no differences in days of hospital stay. No differences were found for sex, functional type, surgical complications, invasiveness, or size **Table 2**.

**Table 2:** Postoperative polyuria.

	Polyuria^1^ (48) n (%)	No alterations^2^ (141) n (%)	p-value^3^
Age at baseline (years) median (IQR)	53 (41-61)	58 (47,5-70)	0.003^4^
Age at diagnosis (years) median (IQR)	49 (38-55)	54 (42-66)	0.005^4^
Hospital stay for surgery (days) median (IQR)	6 (5-7)	5 (4-7)	0.5^4^
Maximum size (mm) median (SD)	21.89 ± 13.71	21.92 ± 11.75	0.986^5^
Sex Male Female			0.433
	19 (39.6)	65 (46.1)	
	29 (60.4)	76 (53.9)	
Functional type Non-functioning ACTH GH			0.131
	26 (54.2)	79 (56)	
	15 (31.3)	27 (19.1)	
	7 (14.6)	35 (24.8)	
Previous visual deficit Yes No NA			0.840
	20 (41.7)	54 (40)	
	28 (58.3)	81 (60)	
		6	
Size^6^ Microadenoma Macroadenoma NA			0.636
	11 (22.9)	27 (19.7)	
	37 (77.1)	110 (80.3)	
		4	
Size > 30 mm Yes No NA			0.651
	11 (22.9)	109 (79.5)	
	37 (77.1)	28 (20.5)	
		4	

IQR, Interquartile range.

SD, Standard deviation.

NA, Data not available.

1. Postoperative polyuria.

2. No water and electrolyte imbalances.

3. Chi-square, unless otherwise specified.

4. Mann-Whitney U.

5. Student’s t test for independent samples.

6. Macroadenoma size ≥ 10 mm.

### Diabetes insipidus

The overall prevalence of DI was 6.89% (14 patients). Among the patients with transient DI, the duration was less than 3 months in three (1.47%) and between 3 and 6 months in two (0.98%). Global mean duration was 2.8 months ± 1,79. Nine patients had permanent DI (4.43%). In the comparative analysis, the patients with DI presented differences in age (p = 0.01) and in days of hospital stay, with a median of 7.5 days for patients with DI compared to 5 days for patients without water and electrolyte disturbance (p = 0.009). There were also differences in the presence of previous visual deficit 71.4% in patients with DI versus 40% in patients without water and electrolyte disturbance (p = 0.044). There were no differences in size, functional type, invasion or complications **Table 3**. In a subanalysis of the two groups, no differences were found between the transient DI group and patients without water and electrolyte disturbance. Patients with permanent DI presented differences in age, days of hospital stay and visual deficit as show in **Table 4**.

**Table 3:** Diabetes insipidus.

	DI^1^ (48) n (%)	Control^2^ (141) n (%)	p-value^3^
Age at baseline (years) median (IQR)	45.5 (40.25-59.5)	58 (47.5-70)	0.010^4^
Age at diagnosis (years) median (IQR)	41 (34.25-55.25)	54 (42-66)	0.008^4^
Hospital stay for surgery (days) median (IQR)	7.5 (5.75-10)	5 (4-7)	0.009^4^
Maximum size (mm) median (SD)	23.8 ± 12.63	21.92 ± 11.75	0.573^5^
Sex Male Female			0.430
	8 (57.1)	65 (46.1)	
	6 (42.9)	76 (53.9)	
Functional type Non-functioning ACTH GH			0.527
	10 (71.4)	79 (56)	
	2 (14.3)	27 (19.1)	
	2 (14.3)	35 (24.8)	
Previous visual deficit Yes No NA			0.044^6^
	10 (71.4)	54 (40)	
	4 (28.6)	81 (60)	
		6	
Size^7^ Microadenoma Macroadenoma NA			0.624
	2 (14.3)	27 (19.7)	
	12 (85.7)	110 (80.3)	
		4	
Size > 30 mm Yes No NA			0.422
	4 (28.6)	109 (79.5)	
	10 (77.4)	28 (20.5)	
		4	

IQR, Interquartile range.

SD, Standard deviation.

NA, Data not available.

1. Diabetes insipidus.

2. No water and electrolyte imbalances.

3. Chi-square, unless otherwise specified.

4. Mann-Whitney U.

5. Student’s t test for independent samples.

6. Fisher’s exact test.

7. Macroadenoma size ≥ 10 mm.

**Table 4:** Permanent Diabetes insipidus.

	PDI^1^ (9) n (%)	Control^2^ (141) n (%)	p-value^3^
Age at baseline (years) median (IQR)	45 (42.5-58)	58 (47.5-70)	0.023
Age at diagnosis (years) median (IQR)	41 (36.5-52.5)	54 (42-66)	0.026
Hospital stay for surgery (days) median (IQR)	7 (5.5-10)	5 (4-7)	0.028^4^
Previous visual deficit Yes No NA			0.005^4^
	8 (88,9)	54 (40)	
	1 (11,1)	81 (60)	
		6	

IQR, Interquartile range.

NA, Data not available.

1. Permanent Diabetes insipidus.

2. No water and electrolyte imbalances.

3. Mann-Whitney U unless otherwise specified.

4. Fisher’s exact test.

## Discussion

EEEA was the surgical technique that was carried out. This allows a greater working angle that provides a better exposure of intrasellar and parasellar lesions (10). Our study shows that water and electrolyte disturbances with polyuria are common in transsphenoidal pituitary surgery. One third of the patients developed a water and electrolyte imbalance.

Regarding the use of desmopressin in postoperative polyuria, 91.7% of the patients treated needed only one or two doses. It was therefore a brief process, which resolved in less than 24 hours, starting between the first and second day after surgery. Only 6.45% of the patients treated with desmopressin developed moderate-severe hyponatremia. It should be noted that half of the patients who were hypernatremic prior to desmopressin administration subsequently developed permanent DI. An initial sodium determination with hypernatremia could thus be a predictor of DI.

Stricter protocols for the treatment of DI during hospitalization are available in the literature, such as the one proposed by *Vries F* et al. (6 *)*, by determining hypotonic polyuria according to the following criteria: diuresis > 300 ml/h for three consecutive hours, a urine density < 1005, and the presence of at least one of the following criteria: excessive thirst, serum osmolarity > 300 mOsmol/kg or serum sodium > 145 mEq/L. However, according to our results, we achieved a simple assessment of polyuria without a negative impact on the patients.

Postoperative polyuria was present in 23.8% of the patients. A wide variety of factors may cause polyuria. The administration of large amounts of perioperative fluids is probably the most frequent cause of polyuria in the immediate postoperative period. Fluid retention depends on the degree of stimulation of the sympathetic system, AVP or aldosterone secretion, and renal flow. In the postoperative period, physiologic hypotonic polyuria occurs with the decrease in stress hormones and hemodynamic stabilization. On the other hand, immediate polyuria is caused by a transient AVP deficiency secondary to decreased secretion by the magnocellular nucleus after surgical manipulation (11).

There is great variability in the literature regarding the prevalence of transient DI after transsphenoidal pituitary surgery, ranging between 5 and 25%. This is presumably due to the different denominations of transient DI, and the inclusion of postoperative physiologic polyuria as part of this condition (11). In our study, the prevalence of transient DI was 2.45%, which could be explained in part by a correct exclusion of postoperative physiologic polyuria. In contrast, the prevalence of permanent DI (4.43%) in our cohort was similar to that described in the literature (1-4%) for endoscopic transsphenoidal surgery (2). Similar rates have also been described for the extended approach (12, 13). The figures for microscopic surgery are as high as 8.8% (2).

In our series, patients with postoperative polyuria did not have a longer hospital stay than those without this type of disorder. Conversely, patients with DI had a longer hospital stay that could not be attributed to other morbidity conditions such as post-surgical complications. This is in agreement with the literature in which several studies have shown that DI increases the duration of hospital stay and the need for readmission (14, 15). All of these factors have a negative economic impact. The identification of the predictive factors for postoperative DI may therefore have a beneficial effect in reducing morbidity. Given that postoperative polyuria does not imply greater morbidity, a clear division between this condition and the different types of DI is needed to determine the true morbidity of these processes.

In our study, it appears that younger patient age at diagnosis and age translate into a greater likelihood of developing postoperative polyuria and DI. While *Ajlan* et al. (16) do not associate younger age with DI, *Hensen* et al. (11) and *Rudolf* et al. (17) do associate younger age with DI. The latter group found this association only with transient DI. It is probable that the findings obtained in younger patients are due to the fact that in these patients more aggressive surgeries are usually performed with greater manipulation or removal of the pituitary gland due to their longer life expectancy. There is often greater manipulation of the pituitary stalk with lesions in the lower pituitary stalk, causing no permanent damage (3).

In our series, previous visual deficit was significantly associated with the development of DI. The visual impairment produced by pituitary adenomas is due to craniocaudal growth with invasion of the suprasellar area, which results in optic nerve damage. Therefore, in the resection of adenomas, in this case and in contrast to what was explained in the previous paragraph, damage at a higher level of the pituitary stalk can occur, which can lead to the development of DI (3, 18, 19).

While size has been linked to an increase in DI (6), in our study there was no significant increase in the risk of DI or postoperative polyuria. Other groups have found a relationship with size, but this was measured craniocaudally, as it was related to suprasellar extension (20, 21). In our analysis we used maximum size, without distinguishing craniocaudal length. However, we also found no differences with tumors larger than 30 mm.

The Knosp classification is the most commonly used system to determine invasiveness and to predict the difficulty and possible complications related to surgery. In our study, as in the literature, this classification does not predict the occurrence of postoperative polyuria, transient DI, or permanent DI (21, 22).

Finally, sex did not appear to predict polyuric disorders, which is consistent with the literature (21). The functional type in our study also did not predict postoperative polyuria, transient DI, or permanent DI, which coincides with the review by *Lobatto* et al. (21), although some authors find an increased risk for the group of ACTH- secreting adenomas in transient DI (20). A specific condition of pituitary tumors producing GH and ACTH hormones has been described in the literature, with these hormones giving rise to sodium and water retention resulting in a negative balance as they decrease following surgery (6). Although this occurs, it is possible that these negative balances are not clinically relevant to the findings of the study. The results of the study may be biased by the size of the sample and a larger study would be needed to evaluate the findings.

In conclusion, water and electrolyte disorders are common after pituitary surgery. Most cases are transient hypotonic polyuria that resolves within one day. The prevalence of DI in our cohort of patients undergoing extended transsphenoidal surgery was lower than that described in the literature, at the expense of transient DI.

## Data availability statement

The raw data supporting the conclusions of this article will be made available by the authors, without undue reservation.

## Ethics statement

Ethical review and approval was not required for the study on human participants in accordance with the local legislation and institutional requirements. Written informed consent for participation was not required for this study in accordance with the national legislation and the institutional requirements.

## Author contributions

JC, ED, and AM designed the study. JC, CM and PR performed data collection. JC, ED, AM analyzed the data. JC wrote the first draft. JC, ED, EV, AM, PR, AP, EC, AK wrote the paper. All authors contributed to the article and approved the submitted version.

## Acknowledgments

The authors thank Maria Repice for her help with the English version of the text.

## Conflict of interest

The authors declare that the research was conducted in the absence of any commercial or financial relationships that could be construed as a potential conflict of interest.

## Publisher’s note

All claims expressed in this article are solely those of the authors and do not necessarily represent those of their affiliated organizations, or those of the publisher, the editors and the reviewers. Any product that may be evaluated in this article, or claim that may be made by its manufacturer, is not guaranteed or endorsed by the publisher.
